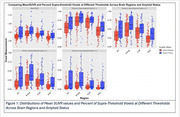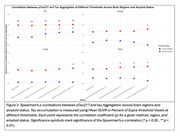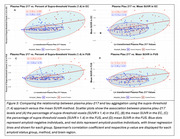# Evaluating Plasma Ptau‐217 Sensitivity in Detecting Individual Differences in Early‐stage Tau Aggregation in Cognitively Unimpaired Older Adults

**DOI:** 10.1002/alz70862_110003

**Published:** 2025-12-23

**Authors:** Leah S Varghese, Lisa M. Taylor, Elizabeth A. Thomas, Michael A Yassa, Jenna N. Adams

**Affiliations:** ^1^ University of California, Irvine, Irvine, CA USA

## Abstract

**Background:**

Plasma ptau‐217 is a promising biomarker for detecting Alzheimer's pathology. However, previous studies show no or weak correlations between plasma ptau‐217 and tau‐PET in cognitively unimpaired (CU) individuals. This is possibly due to limitations in the mean SUVR‐based ROI analysis, which lacks sensitivity to subtle, focal individual differences in early‐stage tau aggregation. We utilized a supra‐threshold voxel‐based approach to re‐examine the relationship between plasma ptau‐217 and early‐stage tau aggregation in the medial temporal lobe (MTL) as a function of amyloid‐beta (Aβ) status in CU older adults.

**Methods:**

We analyzed 142 CU older adults (95 Aβ‐/47 Aβ+) from ADNI with plasma ptau‐217 (Janssen assay) and 18F‐Flortaucipir‐PET data. SUVR thresholds (1.1, 1.2, 1.3, 1.4) were applied to tau‐PET data to calculate the percentage of suprathreshold voxels in entorhinal cortex (EC), parahippocampal gyrus (PHC), amygdala (Amyg), and fusiform gyrus (FUS) (Figure 1). The mean SUVR for each ROI was also calculated for comparison. Spearman's rank correlation assessed relationships between plasma ptau‐217 and regional tau‐PET.

**Results:**

In Aβ‐ participants, the supra‐threshold method (SUVR ≥1.4) in the EC showed the strongest correlation with plasma ptau‐217 (ρ=0.25, *p* = 0.011; Figure 2‐3), while the mean SUVR method showed a weaker, non‐significant correlation (ρ=0.15, *p* = 0.14). In Aβ+ participants, the supra‐threshold method (SUVR ≥1.4) was equivalent to the mean SUVR method in the EC; however, it demonstrated a more sensitive detection of tau‐PET in later‐stage MTL regions such as Amyg, PHC, and FUS, while the mean SUVR method did not reveal significant associations (Figures 2‐3).

**Conclusion:**

The supra‐threshold approach is a more sensitive measure than mean SUVR for detecting early tau aggregation in regions not yet saturated by tau pathology. In CU individuals, plasma ptau‐217 correlates with suprathreshold tau‐PET in the EC even without Aβ. In MTL regions that aggregate tau after EC (PHC/Amyg/FUS), plasma ptau‐217 was again associated with suprathreshold tau‐PET, but not mean SUVR, and this relationship emerges only with Aβ pathology. These results provide insight into the temporal dynamics of Aβ‐related tau pathology and suggest that plasma ptau‐217 can serve as a useful biomarker for early detection of tau accumulation when an appropriately sensitive method is used.